# A scoping review on examination approaches for identifying tactile deficits at the upper extremity in individuals with stroke

**DOI:** 10.1186/s12984-024-01397-8

**Published:** 2024-06-08

**Authors:** Arco P. Paul, Karan Nayak, Lindsey C. Sydnor, Nahid Kalantaryardebily, Kevin M. Parcetich, Daniel G. Miner, Q. Eileen Wafford, Jane E. Sullivan, Netta Gurari

**Affiliations:** 1 Physical Therapy, Radford University, Radford, Virginia USA; 2 Neuroscience, Northwestern University, Evanston, Illinois USA; 3 Neuroscience, Virginia Tech, Blacksburg, Virginia USA; 4 Engineering Mechanics, Virginia Tech, Blacksburg, Virginia USA; 5 Galter Health Sciences Library & Learning Center, Northwestern University, Evanston, Illinois USA; 6 Physical Therapy and Human Movement Sciences, Northwestern University, Chicago, Illinois USA; 7 Biomedical Engineering and Mechanics, Virginia Tech, Blacksburg, Virginia USA

## Abstract

**Purpose:**

Accurate perception of tactile stimuli is essential for performing and learning activities of daily living. Through this scoping review, we sought to summarize existing examination approaches for identifying tactile deficits at the upper extremity in individuals with stroke. The goal was to identify current limitations and future research needs for designing more comprehensive examination tools.

**Methods:**

A scoping review was conducted in accordance with the Joanna Briggs Institute methodological framework and the PRISMA for Scoping Reviews (PRISMA-ScR) guidelines. A database search for tactile examination approaches at the upper extremity of individuals with stroke was conducted using Medline (Ovid), The Cochrane Library (Wiley), CINAHL Plus with Full Text (Ebsco), Scopus (Elsevier), PsycInfo (Ebsco), and Proquest Dissertations and Theses Global. Original research and review articles that involved adults (18 years or older) with stroke, and performed tactile examinations at the upper extremity were eligible for inclusion. Data items extracted from the selected articles included: if the examination was behavioral in nature and involved neuroimaging, the extent to which the arm participated during the examination, the number of possible outcomes of the examination, the type(s) of tactile stimulation equipment used, the location(s) along the arm examined, the peripheral nerves targeted for examination, and if any comparison was made with the non-paretic arm or with the arms of individuals who are neurotypical.

**Results:**

Twenty-two articles met the inclusion criteria and were accepted in this review. Most examination approaches were behavioral in nature and involved self-reporting of whether a tactile stimulus was felt while the arm remained passive (i.e., no volitional muscle activity). Typically, the number of possible outcomes with these behavioral approaches were limited (2-3), whereas the neuroimaging approaches had many more possible outcomes ($>15$). Tactile examinations were conducted mostly at the distal locations along the arm (finger or hand) without targeting any specific peripheral nerve. Although a majority of articles compared paretic and non-paretic arms, most did not compare outcomes to a control group of individuals who are neurotypical.

**Discussion:**

Our findings noted that most upper extremity tactile examinations are behavioral approaches, which are subjective in nature, lack adequate resolution, and are insufficient to identify the underlying neural mechanisms of tactile deficits. Also, most examinations are administered at distal locations of the upper extremity when the examinee’s arm is relaxed (passive). Further research is needed to develop better tactile examination tools that combine behavioral responses and neurophysiological outcomes, and allow volitional tactile exploration. Approaches that include testing of multiple body locations/nerves along the upper extremity, provide higher resolution of outcomes, and consider normative comparisons with individuals who are neurotypical may provide a more comprehensive understanding of the tactile deficits occurring following a stroke.

**Supplementary Information:**

The online version contains supplementary material available at 10.1186/s12984-024-01397-8.

## Introduction

Intact somatosensory perception is essential to interact with our surrounding environment, including when performing and learning skilled movements [1, 2]. Successful execution of voluntary movements depends on accurately processing and perceiving the incoming somatosensory information [3]. Somatosensory impairments following stroke are relatively common, affecting upwards of 85% of survivors living with stroke [4, 5]. Specifically, loss of tactile perception following stroke is a commonly occurring somatosensory deficit amongst survivors [5, 6]. Types of tactile deficits that are commonly seen following stroke include hypoesthesia (reduced ability to feel touch), dysesthesia (abnormal tactile perception), and impaired two-point discrimination (reduced ability to discriminate between two nearby locations of touch) [7, 8]. The presence of tactile deficits post stroke is a negative prognostic factor for upper-limb motor recovery [5, 9], with the ability to discriminate two nearby points of touch on the skin (two-point discrimination) after acute stroke being a good early predictor of dexterous hand function [10]. Most clinicians examine somatosensory modalities, including touch [11], and are aware of the association between somatosensory function and motor recovery. Nevertheless, there is a shortage of rigorous, evidence-based clinical practice guidelines to evaluate and, in turn, treat tactile dysfunction [12–14]. The shortcoming is, in part, due to a limited understanding of the underlying reason for tactile perceptual deficits following a stroke [7].

Tactile stimulation activates mechanoreceptors in the skin. The resulting tactile signals are transmitted along the peripheral nervous system, into the dorsal column medial-lemniscus pathway, and then onwards to the primary and secondary somatosensory cortices where conscious tactile perception occurs. A common clinical approach for examining tactile dysfunction post stroke utilizes behavioral tests that have subjective measurements [15, 16]. These tests require an individual to indicate when and where a stimulus is felt. Such tests enable the examiner to determine whether a tactile stimulus is perceived. However, these behavioral tests do not elucidate the anatomical and physiological nature of disruption(s) in tactile perceptual pathways of the nervous system. On the other hand, the integrity of the tactile systems can also be examined by using neurophysiological tools such as neuroimaging and recordings of electrical activity along the nervous system following tactile stimulation [7, 17]. Such tools can indicate whether a tactile stimulus delivered at the periphery travels effectively along the nervous system and reaches an individual’s brain. Yet, common approaches for tracking tactile signals along the nervous system typically report activity within the brain and, hence, may not identify the exact location(s) along the somatosensory pathways where signal transmission is disrupted following a stroke.

In this scoping review, we aimed to summarize the existing approaches and tools for examining tactile deficits at the arm post stroke. We focused on deficits aligned with increased conscious detection thresholds for perceiving touch (e.g., hypoesthesia), abnormal tactile perception (e.g., dysesthesia), and impaired two-point discrimination. The goal was to review examination tools that measure these tactile deficits and provide insights into the underlying neural mechanisms eliciting the deficits. Through this process, we aimed to summarize limitations in existing examination approaches and identify future research needs for designing more precise tactile examination protocols. In turn, such research endeavors could lead to the development of improved tactile intervention strategies that improve sensorimotor outcomes in stroke rehabilitation.

## Methods

The Joanna Briggs Institute methodological framework (https://jbi.global/scoping-review-network/resources) and the Preferred Reporting Items for Systematic Reviews and Meta-Analyses extension for Scoping Reviews (PRISMA-ScR) [18] were used to design this review. This study employed the Population-Concept-Context framework [19] to identify main elements for the search. The population included individuals who have experienced a stroke. The concept included approaches used to examine tactile deficits at the upper extremity in this population, and the context included examinations performed on individuals with stroke in the clinical and research settings. This scoping review protocol is registered on DigitalHub within the Galter Health Sciences Library & Learning Center at Northwestern University (https://prism.northwestern.edu/records/ys72z-dgx56).

### Search strategy

A research librarian (QEW) developed a comprehensive search strategy that incorporated keywords and Medical Subject Heading (MeSH) terms describing individuals with stroke, upper extremities, examinations, and tactile deficits. Examples of terms used for searches included words associated with stroke (e.g., cerebrovascular accident, ischemia, embolism, intracranial, infarct), body parts of the upper extremity (e.g., hand, forearm), tactile signaling and perception (e.g., touch, pressure, vibration, cutaneous, skin, sensation, perception, haptic), and examination (e.g., assessment, evaluation). These words were selected to identify studies that used examination tools to characterize tactile signaling and perception at the upper extremity of individuals with stroke. The following databases were searched from the date of inception to the date of the search (August 18, 2022): Medline (Ovid), The Cochrane Library (Wiley), CINAHL Plus with Full Text (Ebsco), Scopus (Elsevier), PsycInfo (Ebsco), and Proquest Dissertations and Theses Global. The search was limited to studies of the human population and English publications. No restrictions on publication date or research design were applied. The full search strategy can be accessed in the supplementary material provided with this scoping review.

### Eligibility criteria

Original research articles (e.g., controlled trials, cohort studies, case reports) that enrolled adult human participants (18 years or older) with any type of stroke, and performed tactile examinations at the upper extremity were eligible for inclusion. Articles that examined individuals with conditions other than stroke, examined non-tactile deficits (e.g., proprioception, pain, temperature), examined deficits that involved additional cognitive areas and complex processing (e.g., stereognosis, unilateral spatial neglect, tactile extinction), or involved a population aged under 18 were excluded from this review. Systematic reviews, opinion reviews, and scoping reviews were excluded.

### Article selection process

The article selection was conducted using a two-step process - screening titles/abstracts for all articles, followed by screening the full text of the shortlisted articles.

For this process, 2 reviewers (KN, LS) were trained to screen articles by 3 content experts (AP, KP, DM). These 2 reviewers were first trained on the title/abstract screening process. The 2 reviewers independently screened the titles/abstracts of the first 100 articles that were returned from the database searches. After screening, they met to discuss which articles they included and excluded. The target was > 80% agreement on the decision to include or exclude articles. For articles with conflicting decisions, the 2 reviewers discussed their results with the 3 content experts to obtain an improved understanding/consensus on how to screen the articles. After achieving > 80% agreement, the 2 reviewers were trained by the content experts to perform the full-text screening using a sample of 3 representative articles. Once the title/abstract and full-text screening training was successfully completed, the 2 reviewers were approved to conduct the title/abstract and full-text screening of all the articles.

After screening the titles and abstracts, the 2 trained reviewers met to discuss and compile the results of the articles to include and exclude. For articles in which a conflict arose, the decision of whether to include or exclude the article was resolved with the 3 content experts. Next, the 2 trained reviewers independently reviewed the full text of all the included articles to further assess their eligibility for inclusion. Any conflicts were resolved again with the 3 content experts.

### Data extraction

The included articles were divided amongst pairs consisting of 1 content expert and 1 trained reviewer to extract data items of interest. Each pair worked together to resolve any conflicts that arose in the data extraction process. Extracted data included:

- Tactile Examination Approach (e.g., behavioral, neuroimaging)
- Type of Arm Participation during Examination (e.g., volitional movement, passive state, active-assist)
- Number of Possible Examination Outcomes
- Tactile Examination Equipment
- Body Location of Examination (e.g., elbow, fingertip)
- Peripheral Nerve Targeted for Examination (e.g., median, ulnar, radial)
- Laterality of Examination (paretic arm, non-paretic arm, both)
- Comparison to Arm(s) of Individuals who are Neurotypical (none, one arm, both arms)

**Fig. 1:**
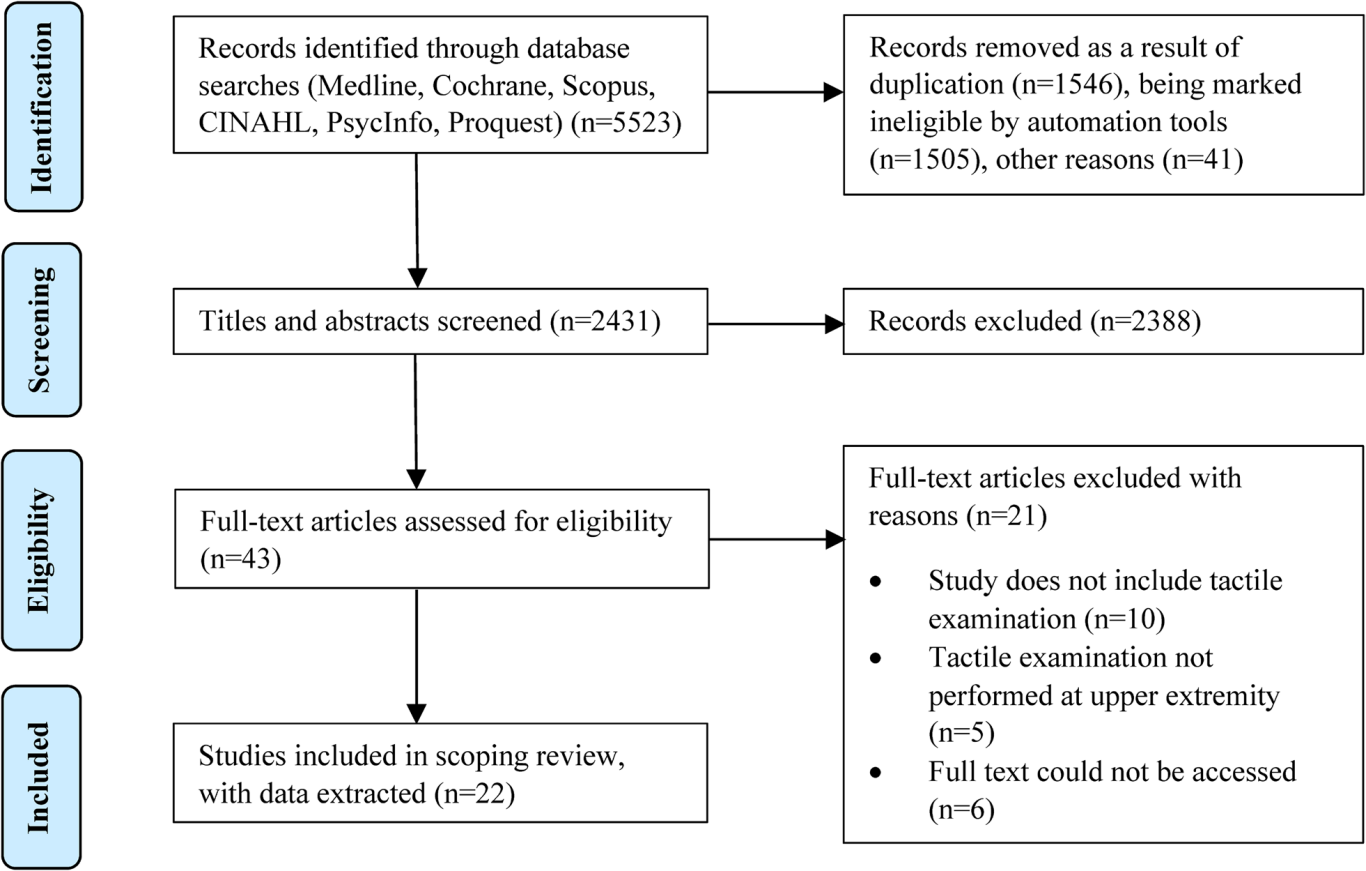
PRISMA flow diagram showing the article selection process

### Data synthesis

To synthesize the data, we captured the number of articles that identified the different items of interest for tactile examination, as indicted in the ‘Data extraction’ section above. Common themes were generated from the collated data.

## Results

An overview of the PRISMA-ScR article selection process is provided in Fig. 1. The original search identified 5,523 articles. After removing records for reasons including duplication and being marked ineligible by automation tools, 2,431 articles remained for consideration. Of those articles, 2,388 were removed following the title/abstract screening. The remaining 43 articles underwent a full-text review, resulting in 21 more articles being excluded. Upon completion of the selection process, 22 articles were included in this review. The included articles and extracted data are summarized in Tables 1 and 2.

**Table 1:** Summary of Examination Approaches

References	Examination approach	Type of arm participation during examination	Number of possible outcomes	Tactile examination equipment
Zhou et al. 2021 [20]	Neuroimaging	Passive	>15	Cotton Fabric
Villepinte et al. 2019 [15]	Behavioral	Passive	3	Cotton Wool, Experimenter’s Index Finger, Two-Point Discriminator
Kessner et al. 2019 [6]	Behavioral	Passive	2	Cotton Swab
Mandehgari et al. 2018 [21]	Behavioral	Passive	>15	Von Frey Monofilaments, Two-Point Discriminator
Boccuni et al. 2018 [22]	Behavioral	Passive	3	Cotton Wool, Experimenter’s Index Finger
Ballardini et al. 2018 [23]	Behavioral	Passive	>15	Skin Brush Robotic Stimulator
Meyer et al., 2016 [24]	Behavioral	Passive	3, >15	Cotton Wool, Experimenter’s Index Finger, Electrotactile Stimulator
Lima et al. 2015 [25]	Behavioral	Passive	3, 7	Von Frey Monofilaments, Fabric, Cotton Wool, Experimenter’s Index Finger
Bowden et al. 2014 [26]	Behavioral	Passive	NR	Von Frey Monofilaments
Jang et al. 2013 [27]	Behavioral	Passive	3	Cotton Ball, Experimenter’s Index Finger
Jang and Lee 2013 [28]	Behavioral, Neuroimaging	Passive	3, >15	Brush
Sullivan et al. 2011 [29]	Behavioral	Passive	3	Cotton Ball
Michaelsen et al. 2011 [30]	Behavioral	Passive	3	Cotton Wool
Hedman and Sullivan 2011 [31]	Behavioral	Passive	3, >15	Electrotactile Stimulator, Cotton Ball
Connell et al. 2008 [5]	Behavioral	Passive	3	Cotton Wool, Experimenter’s Index Finger, Neurometer
Welmer et al. 2008 [32]	Behavioral	Passive	2	Cotton Wool
Lin et al. 2004 [33]	Behavioral	Passive	3	Cotton Wool
Damyanovich and Orlova 2004 [34]	Neuroimaging	Passive	>15	Electrotactile Stimulator
Druschky et al. 2002 [35]	Neuroimaging	Passive	>15	Automated Pneumatic Stimulator
Dannenbaum et al. 2002 [36]	Behavioral	Passive and Active	3, 2	Brush, Ball
Carey et al. 1997 [37]	Behavioral	Active	15	Graded Plastic Textures
Chiang and Chiu 1989 [38]	Neuroimaging	Passive	>15	Electrotactile Stimulator

Details are provided for each article regarding whether tactile deficits were determined using a behavioral versus neuroimaging examination, whether during the examination the arm volitionally activated (Active) or not (Passive), the number of possible outcomes to define the extent of the tactile deficit, and the tactile equipment used for the examination.

*NR* Not Reported

**Table 2:** Summary of Body Locations, Nerves, and Comparisons used for Examination

Reference	Body location of examination	Targeted nerve	Laterality of examination in stroke	Comparison to arms of individuals who are neurotypical
Zhou et al. 2021 [20]	Forearm	NR	Paretic, Non-Paretic	Both Arms
Villepinte et al. 2019 [15]	Upper Arm, Forearm, Hand, Finger	NR	Paretic, Non-Paretic	NR
Kessner et al. 62019 []	Shoulder, Hand	NR	Paretic, Non-Paretic	Both Arms
Mandehgari et al. 2018 [21]	Hand, Finger	NR	Paretic	NR
Boccuni et al. 2018 [22]	Shoulder, Upper Arm, Elbow, Forearm, Wrist, Hand, Finger	NR	Paretic	NR
Ballardini et al. 2018 [23]	Hand	NR	Paretic, Non-Paretic	Both Arms
Meyer et al. 2016 [24]	Finger	NR	Paretic	NR
Lima et al. 2015 [25]	Hand, Wrist	NR	Paretic, Non-Paretic	Both Arms
Bowden et al. 2014 [26]	Hand	Median, Ulnar, Radial	Paretic, Non-Paretic	Both Arms
Jang et al. 2013 [27]	NR	NR	Paretic, Non-Paretic	NR
Jang and Lee 2013 [28]	Finger	NR	Paretic	Both Arms
Sullivan et al. 2011 [29]	Upper Arm, Forearm, Hand	NR	Paretic, Non-Paretic	NR
Michaelsen et al. 2011 [30]	Shoulder, Upper Arm, Forearm, Finger	NR	Paretic, Non-Paretic	NR
Hedman and Sullivan 2011 [31]	Finger	NR	Paretic, Non-Paretic	NR
Connell et al. 2008 [5]	Shoulder, Elbow, Wrist, Hand	NR	Paretic, Non-Paretic	NR
Welmer et al. 2008 [32]	Upper Arm, Forearm, Hand	NR	Paretic	NR
Lin et al. 2004 [33]	Hand	NR	Paretic, Non-Paretic	NR
Damyanovich and Orlova 2004 [34]	Wrist	Median	Paretic, Non-Paretic	One Arm
Druschky et al. 2002 [35]	Finger	NR	Paretic, Non-Paretic	Both Arms
Dannenbaum et al. 2002 [36]	Hand, Finger	NR	Paretic, Non-Paretic	NR
Carey et al. 1997 [37]	Finger	NR	Paretic, Non-Paretic	One Arm
Chiang and Chiu 1989 [38]	Wrist	Median	Paretic	NR

Details are provided for each article regarding body locations on the arm examined, the specific nerve(s) examined, whether the paretic and non-paretic arm were assessed (Laterality of Examination), and whether a comparison was included to either or both arms of individuals who are neurotypical (Comparisons to Arms of Individuals who are Neurotypical).

*NR* Not Reported

**Fig. 2:**
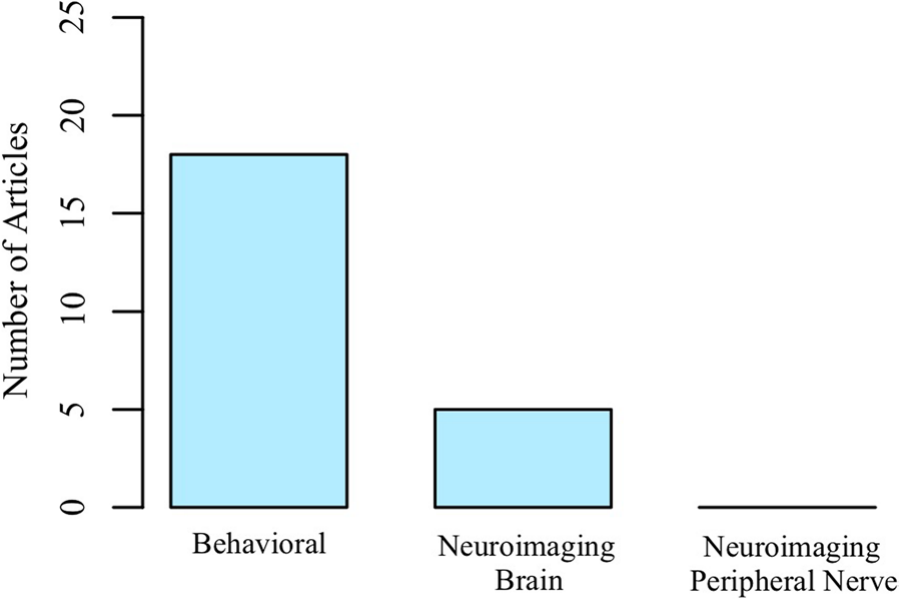
Type of Examination Approach.The majority of the tactile examination approaches used behavioral testing, and a few used neuroimaging of the brain

An outcome of consideration was whether a behavioral approach and/or a neurophysiological approach was used for the tactile examination since neurophysiological approaches can provide additional insights into the inner workings of the tactile systems. Figure 2 summarizes the distribution of examination approaches used. Most articles used a behavioral approach (n=18), whereas only a handful used a brain neuroimaging approach (n=5). Only 1 of the 22 articles included both a behavioral approach and a brain neuroimaging approach to evaluate tactile signaling and perception post stroke [28]. None of the articles sought to capture the integrity of neural information passed along the peripheral nervous system.

**Fig. 3:**
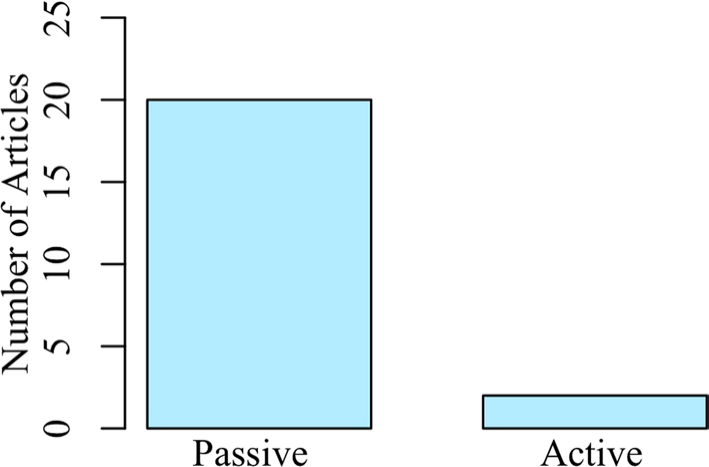
Type of Arm Participation during Examination. 20 studies used an examination approach where the participant’s arm was relaxed (passive), and only 2 studies included volitional muscle activation (active)

Another area of interest was whether participants volitionally activated their arms as part of the examination process since volitional muscle activation can impact how tactile signals are processed and perceived [39, 40]. Only 2 studies used an examination approach which involved active interaction of the participants with a tactile stimulus. In these examinations, participants actively held an object with their fingers [36] or explored graded textures [37]. As shown in Fig. 3, 20 out of the 22 studies used tactile examinations that were designed to detect tactile deficits when the participant was at rest (i.e., no volitional muscle activation at the paretic arm).

**Fig. 4:**
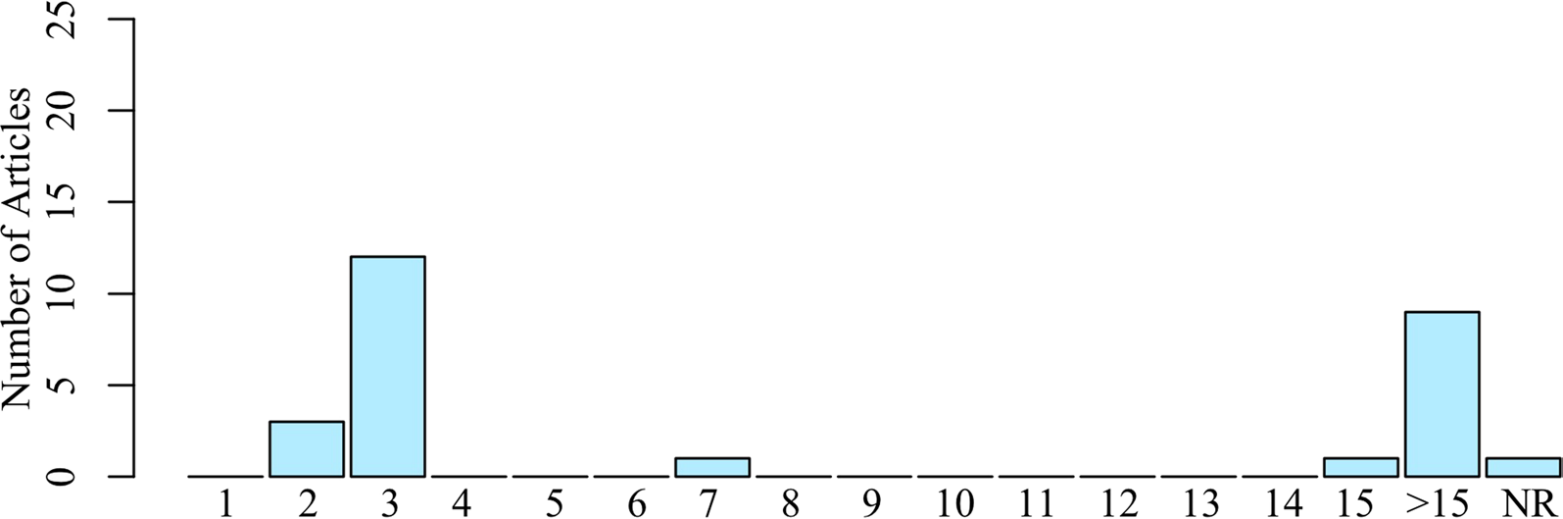
Possible Number of Outcomes of Examination. The number of possible outcomes of the different examination approaches ranged from poor (2-3 possible outcomes) to excellent (>15 possible outcomes). NR: Not Reported

A crucial aspect of an examination is the number of possible outcomes. Too few outcomes can lead to insufficient ability to detect changes in tactile deficits for reasons including poor resolution, inadequate responsiveness, floor effects (inability to detect changes at low magnitudes), and ceiling effects (inability to detect changes at high magnitudes). The behavioral approaches typically had 3 possible subjective outcomes (i.e., intact, impaired, absent), and were determined based on participants’ responses to whether a tactile stimulus, like cotton, a clinician’s finger, or monofilaments, were perceived. One of the behavioral approaches used a robotic device with a high-resolution sensor, enabling a greater number of possible outcomes [23]. The neuroimaging examination approaches (e.g., fMRI, EEG, MEG) all had many possible outcomes. We summarized the possible number of outcomes for the examination approaches used in Fig. 4.

**Fig. 5:**
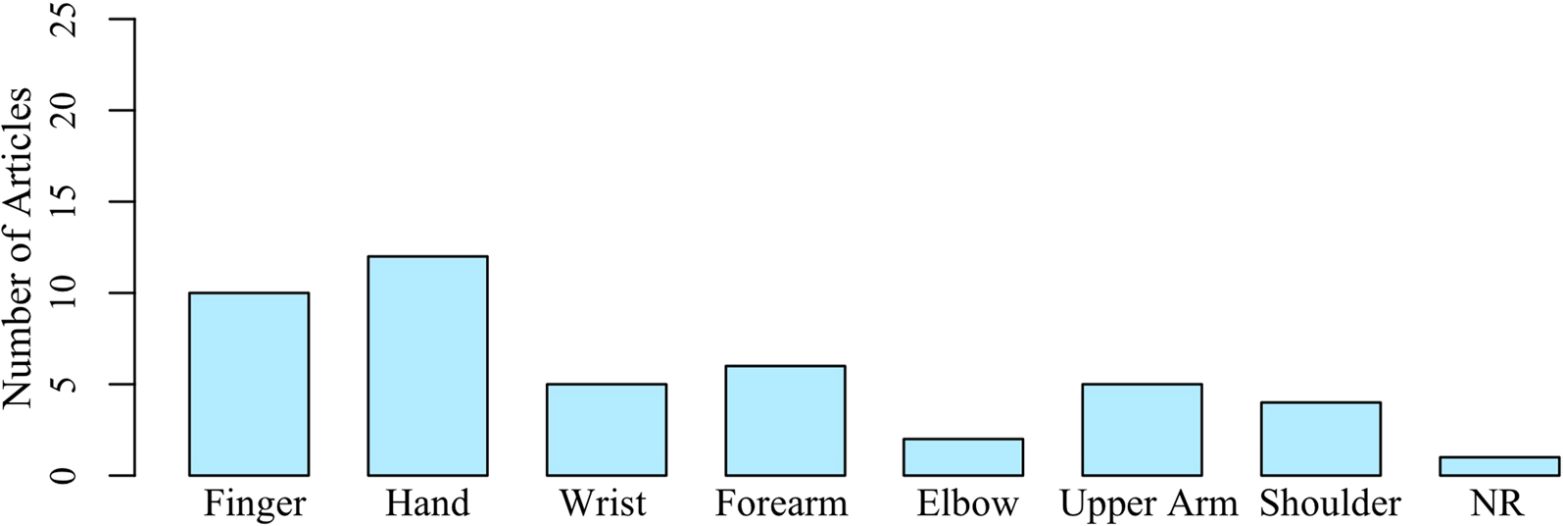
Locations along the Arm that were Examined. A majority of the articles examined at the distal locations of the finger and hand, when compared to more proximal locations at the upper arm or shoulder. NR: Not Reported

**Fig. 6:**
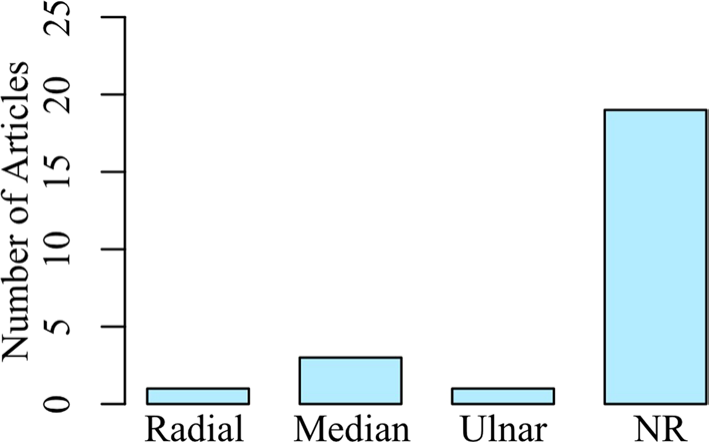
Nerves Reported to be Examined. Most articles did not specifically examine or report the nerves that were examined. NR: Not Reported

We were also interested in exploring the body locations along the arm used during the tactile examination. The common understanding is that deficits are more severe at more distal locations. As shown in Fig. 5, a majority of articles examined tactile dysfunction at distal locations, such as the finger and hand (n=18). Articles that included examination of proximal sites, such as the shoulder, were much less in number (n=4). Some of the articles comprehensively examined both distal and proximal locations, as summarized in Table 2 (n=8). However, 19 of the 22 articles did not specify the peripheral nerves that were targeted for examination. Only 1 article indicated all nerves that were examined [26]. Figure 6 summarizes the number of articles that mentioned specific nerves examined.

**Fig. 7:**
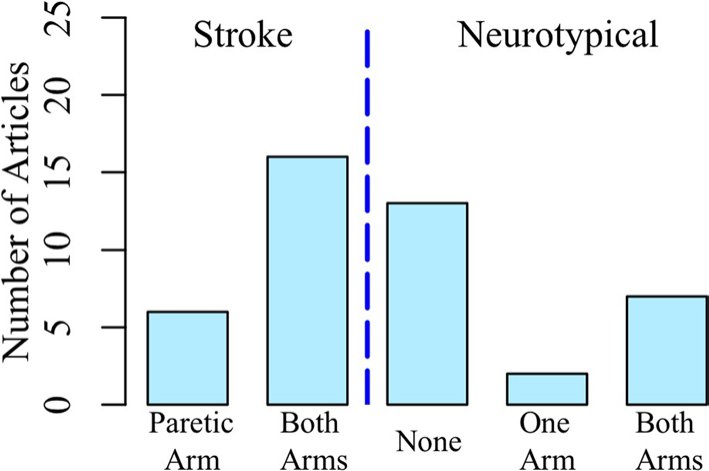
Arm(s) Examined in the Individuals with Stroke and Individuals who are Neurotypical. Although many studies compared the findings of the paretic arm to the non-paretic arm, most did not compare tactile deficits of the individuals with stroke to individuals who are neurotypical

Since the non-paretic arm could be affected in stroke, an important objective of tactile examinations post stroke is to assess tactile deficits at both limbs. Therefore, we captured whether examinations were performed at both arms of individuals with stroke. We also considered if the examination approaches included comparisons with individuals who are neurotypical, such that the extent of the deficits at each arm of individuals with stroke could be determined through comparison. We found that 6 articles did not compare tactile deficits at the paretic arm to the non-paretic arm following stroke. Additionally, a majority of the articles did not compare tactile deficits of individuals with stroke to individuals who are neurotypical (n=13). Figure 7 summarizes the number of articles that compared tactile deficits in the paretic arm to the non-paretic arm of individuals with stroke, and tactile deficits in individuals with stroke to individuals who are neurotypical.

## Discussion

In this review, we summarized tactile examinations currently used for assessing deficits in tactile signaling and perception that are commonly seen in the upper extremity following stroke (e.g., hypoesthesia, impaired two-point discrimination). Features of interest included whether the examination tools used behavioral or neurophysiological approaches, type of arm participation, possible number of outcomes, location(s) along the arm that were tested, and if peripheral nerves were reported to be tested. The goal of this review was to analyze the utility of the currently used approaches for detecting, measuring, and providing insights into the underlying neural mechanisms eliciting tactile signaling and perceptual deficits. We propose that the following aspects of tactile examination warrant further research to fulfill gaps in our understanding of the nature of tactile deficits occurring at the arm after a stroke.

### Examination approach

Tactile perception depends on the integrity of complex processing at various levels along the somatosensory pathway, resulting in a perceived feeling. Given the processing complexity, we recommend that a more complete understanding of tactile perception be obtained by using tactile examination protocols that include both behavioral and neuroimaging approaches. 18 of the 22 articles relied on a behavioral approach for tactile examination, whereas only 1 article included both a behavioral and a neuroimaging approach.

Behavioral approaches require an individual to indicate their experience when brief tactile stimuli are applied to targeted body locations by objects like cotton tips, brush fibers, nylon monofilaments, and the examiner’s fingertip. As an example of a simple clinical tactile examination, a cotton tip can be placed briefly on the skin of the distal end of the index finger of an individual whose eyes are closed. The individual is asked to verbally respond indicating when the cotton tip is felt at that location. Such brief touches can be provided at various body locations during an examination session. Body locations can be specifically chosen to assess the integrity of the tactile signals carried by a specific peripheral nerve or a dermatome of a limb. For example, touch signaling from the distal index finger is used to assess the integrity of the tactile pathways carried via the median nerve and C6 dermatome. A lack of a verbal response could lead a clinician to suspect hypoesthesia in these tactile pathways, a common problem after stroke. Just like any tool, this test has limitations and makes certain assumptions. This behavioral examination approach assumes that the cognitive functions of an individual are intact for accurate verbal responses, which may not be valid. Additionally, this approach relies on an individual to respond whether the touch applied by an examiner is felt; yet, the touch simuli are inherently variable for reasons including differences in timing and pressure that the examiner applies. On the other hand, neurophysiological measurement approaches do not depend on a person’s verbal response, and instead rely on recording the neurophysiological responses following tactile stimulation.

An approach that generates behavioral responses and neurophysiological outcomes, ideally simultaneously, may result in a better understanding for the reason(s) that conscious tactile perceptual deficits occur. Also, we propose an approach that includes tools that can image the structure and function of the peripheral nerves, in addition to the central nervous system. Studies have suggested that changes may occur in peripheral nerve function post stroke [41–44], which could contribute to tactile deficits. Information about the function of the peripheral nerves is potentially instrumental in localizing site(s) along the nervous system that might be responsible for tactile dysfunction in individuals with stroke and could be useful for determining prognosis and intervention strategies.

### Active participation during examination

Performing daily physical tasks, such as cutting food and driving, involves volitional activation of one’s arms. It is known that the quality of tactile perception varies depending on the level of volitional activation that an individual engages in when exploring objects in their environment [39, 40]. Electroencephalography studies have suggested differences in brain activation patterns between passive and active tactile exploration [45, 46]. Even so, only 2 of the 22 articles utilized active physical involvement from participants during examination [36, 37]. Development of examination approaches that include volitional activation of the arm may result in a greater understanding of the effects of tactile deficits during real-life interactions with objects and could lead to more targeted intervention strategies. Although volitional movements could be impaired post stroke due to the brain injury, we propose that efforts be put on designing tactile examination tools that allow for active movements of the arm. In this way, the findings could be more relevant with the nature of tactile deficits experienced in real life when performing daily activities.

### Resolution of examination tool

A concern with existing tactile examination approaches for adults with neurological disorders is their resolution [12]. A limited number of possible outcomes of an examination leads to poor resolution, and ability to discriminate change, which could make it difficult to capture progress/worsening of tactile deficits following a stroke. Behavioral examinations typically had poor resolution (i.e., 2-3 possible outcomes). One reason for the poor resolution is limitations of the equipment used to deliver the tactile stimuli. Tactile stimuli instruments, such as an examiner’s finger, cotton material, and Von Frey monofilaments, are limited in the range of tactile intensities that can be delivered. Automated protocols that deliver physical or electrotactile stimuli in a graded manner can provide a broader range of stimuli and better resolution [12, 47, 48]. Having systems that deliver and measure tactile stimuli with a high resolution and along a large range is beneficial for capturing changes that occur during stroke recovery.

### Comprehensiveness of examination

An overall goal of a tactile examination tool is to comprehensively elucidate the nature, extent, and neural basis of tactile deficits following a stroke. This goal can be achieved by examining tactile deficits at different body locations and nerves along the somatosensory system for both the paretic arm and non-paretic arm in stroke. Deficits are thought to be more severe at distal locations than proximal locations and, hence, the majority of the studies focused on examinations at the distal locations. Even so, a small number of the selected studies included approaches that comprehensively examined both distal and proximal locations, as summarized in Table 2. Despite being comprehensive in testing numerous locations along the arm, these articles did not discuss the nerves that were targeted for examination. It is feasible that quality and patterns of tactile dysfunction differ depending on the nerve targeted for examination. Importantly, it has been shown that the non-paretic arm, in addition to the paretic arm, experience tactile deficits following a stroke [49, 50]. Hence, both arms should be examined for tactile dysfunction. Since the non-paretic arm is not a good comparison for capturing the extent of deficits at the paretic arm, individuals with stroke should be compared to individuals who are neurotypical to determine the extent of the deficits. Given the potential of arm dominance impacting tactile perception, the dominance of the arm examined should also be considered when making such comparisons.

### Potential implications

One target audience for this work is neuroengineers. For this group of individuals, the scoping review may inspire the development of new devices to study tactile signaling and perception post stroke. Such devices would be beneficial given that they would be less dependent on the subjective nature of current examination procedures. New devices can enable examination when cutaneous mechanoreceptors are activated during gradual increases and decreases in stimulation parameters, including in magnitude and texture, to provide better resolution for detecting changes in tactile deficits. An example of one such device is the robotic stimulator created by Ballardini et al., which can create the feeling of the skin being brushed and stretched [23]. Future devices could include tactile stimulation during active exploration of tactile environments.

Another target audience is healthcare research professionals. As mentioned in a recent systematic review, commonly used behavioral tactile examination approaches are subjective, have limited resolution, and lack active exploration of tactile environments [12]. While acknowledging these ongoing limitations, the authors of the systematic review concluded that examination approaches which use electrotactile and monofilament stimuli are the most highly recommended. The rationale for recommending these two approaches was that the stimulation is more reliable and the measurement error is smaller in comparison to other existing approaches. In addition to these considerations, we encourage clinician researchers to develop examination approaches that promote active tactile exploration and minimize examiner input, with the overall goal of understanding the real-world functional impact of tactile deficits.

### Limitations

A potential caveat for researchers when developing precise and comprehensive examination tools is to consider the clinical utility, if intended for clinical translation. Potential barriers, such as the amount of training required, resources involved, and time needed to administer examinations, could be considered when developing such tools.

## Conclusions

In conclusion, our findings from this scoping review noted that most currently used tactile examination tools employ behavioral approaches, which are subjective in nature, lack adequate resolution, and are insufficient to identify the underlying neural mechanisms of tactile deficits. Neurophysiological examination approaches are more automated and quantifiable and may provide insights into the neural mechanisms. Nevertheless, most behavioral and neurophysiological examinations administer tests when a participant’s arm is relaxed, rather than actively engaged in exploring tactile environments. Therefore, these approaches may not reflect the real-world tactile challenges faced by individuals with stroke. Also, the tactile examinations are mostly administered at distal locations in the affected upper extremity (finger and hand), and the results are often not compared with a population that is neurotypical. We propose that further research is needed to develop better tactile examination tools that involve both behavioral and neurophysiological testing, while allowing active tactile exploration. Approaches that include testing of multiple body locations and nerves along the upper extremity, provide higher resolution and range in terms of outcomes, and consider normative comparisons with individuals who are neurotypical may provide a more comprehensive understanding of the tactile deficits following stroke. Future work can also explore more complex cognitive processes including tactile stimuli, such as loss of feeling at one arm during bilateral simultaneous touch (tactile extinction), feeling stimuli on the opposite hand that is not stimulated (mirror touch), and inability to comprehensively interpret tactile stimuli to identify physical objects (stereognosis).

### Supplementary Information

Supplementary file 1.

Supplementary file 2.

## Data Availability

All data generated or analyzed during this study are included in this published article. No datasets were generated or analysed during the current study.
