# Supplementary material for: A scoping review on examination approaches for identifying tactile deficits at the upper extremity in individuals with stroke
Source: J Neuroeng Rehabil. 2024 Jun 8;21:99. doi: 10.1186/s12984-024-01397-8 (PMC11162071; doi:10.1186/s12984-024-01397-8)
Supplement: Supplementary file 2 [file 12984_2024_1397_MOESM2_ESM.pdf]

Database Search Overview

Database	Coverage	Date Searched	Results
Ovid MEDLINE(R) and Epub Ahead of Print, In-Process, In-Data-Review & Other Non-Indexed Citations, Daily and Versions 1946 to August 17, 2022	1946 to present	8/18/2022	868
Cochrane Database of Systematic Reviews Issue 8 of 12, August 2022 (Wiley)	1995 to present	8/18/2022	33
Cochrane Central Register of Controlled Trials Issue 7 of 12, July 2022 (Wiley)	N/A	8/18/2022	490
Scopus (Elsevier)	1788 to present	8/18/2022	1633
CINAHL Plus with Full Text (EBSCOhost)	1937 to present	8/18/2022	291
PsycInfo (EBSCOhost)	1800s to present	8/18/2022	511
ProQuest Dissertation & Theses Global	1939 to present	8/18/2022	151
Total			3977
Total After De-Duplication<br>• Remove using automation (n=2390)<br>• Removed after manual review (n=41)			2431

We searched the databases listed above on August 18, 2022. Records from each database were exported to EndNote. Duplicates were removed through the "Find Duplicates" function within EndNote and manual review. Search strategies from each of the bibliographic databases are available below.

Ovid MEDLINE® and Epub Ahead of Print, In-Process, In-Data-Review & Other Non-Indexed Citations, Daily and Versions 1946 to August 17, 2022
Search Strategy:

#	Searches	Results
1	exp Stroke/	162465
2	(apople* or "cerebral accident*" or "cerebrovascular accident*" or poststroke or stroke or strokes).ti,ab.	301297
3	((brain or cerebral or intracranial or intracerebral) adj2 (bleed* or embolism* or hemiparesis or hemorrhage* or infarct* or infract* or injur* or isch?emi* or thrombo* or "vascular accident*")).ti,ab.	184895
4	1 or 2 or 3	467611
5	exp Upper Extremity/	181948
6	(arm or arms or axilla* or elbow* or finger* or forearm* or hand or hands or metacarpus or shoulder* or thumb* or "upper extremit*" or "upper limb*" or wrist*).ti,ab.	998607
7	5 or 6	1048030

8	exp Touch/	17640
9	exp Skin/	241204
10	*Perception/	17800
11	exp Touch Perception/	4819
12	8 or 9 or 10 or 11	277992
13	exp *"Diagnostic Techniques and Procedures"/	2637154
14	exp Patient Outcome Assessment/	18928
15	exp Disability Evaluation/	55912
16	exp Physical Examination/	1332189
17	exp Symptom Assessment/	7035
18	exp "Surveys and Questionnaires"/	1180048
19	investigative techniques/	0
20	exp Somatosensory Disorders/	24125
21	exp Sensory Thresholds/	58243
22	exp Hemiplegia/	11879
23	13 or 14 or 15 or 16 or 17 or 18 or 19 or 20 or 21 or 22	4521316
24	12 and 23	40594
25	((touch* or tactile or cutaneous* or skin or haptic* or sensation* or sense or senses or sensory or somatosensory or perception*) adj6 (deficit* or hemiplegia* or impairment* or monoplegia* or dysfunction or dysfunctions or assessment* or detect* or evaluation* or examination* or index or indexes or instrument or instruments or measure* or outcome* or questionnaire* or scale or scales or score or scores or survey or surveys or test or tests)).ti,ab.	182086
26	clinical assessment*.ti. and (touch* or tactile or cutaneous* or skin or haptic* or sensation* or sense or senses or sensory or somatosensory or perception* or pressure or vibration* or force).ti,ab.	528
27	(pressure assessment not "blood pressure").ti,ab.	219
28	25 or 26 or 27	182705
29	24 or 28	215398
30	4 and 7 and 29	949
31	exp animals/ not humans/	5037553
32	30 not 31	927
33	limit 32 to english language	868

Cochrane Library

ID	Search Hits
#1	MeSH descriptor: [Stroke] explode all trees	11632
#2	(apople* OR "cerebral accident*" OR "cerebrovascular accident*" OR poststroke OR stroke OR strokes):ti,ab,kw	66446

#3 ((brain OR cerebral OR intracranial OR intracerebral) NEAR/2 (bleed* OR embolism* OR hemiparesis OR hemorrhage* OR infarct* OR infract* OR injur* OR isch?emi* OR thrombo* OR "vascular accident*")):ti,ab,kw 25051
#4 #1 OR #2 OR #3 80492
#5 MeSH descriptor: [Upper Extremity] explode all trees 8066
#6 (arm OR arms OR axilla* OR elbow* OR finger* OR forearm* OR hand OR hands OR metacarpus OR shoulder* OR thumb* OR "upper extremit*" OR "upper limb*" OR wrist*):ti,ab,kw 199423
#7 #5 OR #6 199640
#8 MeSH descriptor: [Touch] explode all trees 684
#9 MeSH descriptor: [Skin] explode all trees 4740
#10 MeSH descriptor: [Perception] this term only 1689
#11 MeSH descriptor: [Touch Perception] explode all trees 132
#12 #8 OR #9 OR #10 OR #11 7155
#13 MeSH descriptor: [Diagnostic Techniques and Procedures] explode all trees 245832
#14 MeSH descriptor: [Patient Outcome Assessment] explode all trees 1394
#15 MeSH descriptor: [Disability Evaluation] explode all trees 3894
#16 MeSH descriptor: [Physical Examination] explode all trees 98387
#17 MeSH descriptor: [Symptom Assessment] explode all trees 307
#18 MeSH descriptor: [Surveys and Questionnaires] explode all trees 58950
#19 MeSH descriptor: [Investigative Techniques] this term only 0
#20 MeSH descriptor: [Somatosensory Disorders] explode all trees 1158
#21 MeSH descriptor: [Sensory Thresholds] explode all trees 3271
#22 MeSH descriptor: [Hemiplegia] explode all trees 804
#23 #13 OR #14 OR #15 OR #16 OR #17 OR #18 OR #19 OR #20 OR #21 OR #22 284995
#24 #12 AND #23 3511
#25 (touch* or tactile or cutaneous* or skin or haptic* or sensation* or sense or senses or sensory or somatosensory or perception*) NEAR/6 (deficit* or hemiplegia* or impairment* or monoplegia* or dysfunction or dysfunctions or assessment* or detect* or evaluation* or examination* or index or indexes or instrument or instruments or measure* or outcome* or questionnaire* or scale or scales or score or scores or survey or surveys or test or tests) 43608
#26 (clinical assessment*):ti AND (touch* or tactile or cutaneous* or skin or haptic* or sensation* or sense or senses or sensory or somatosensory or perception* or pressure or vibration* or force):ti,ab,kw 308
#27 ("pressure assessment*"):ti,ab,kw NOT ("blood pressure"):ti,ab,kw 37
#28 #25 or #26 or #27 43862
#29 #24 OR #28 45402
#30 #4 AND #7 AND #29 523

Scopus

(((TITLE-ABS(apople* OR "cerebral accident*" OR "cerebrovascular accident*" OR poststroke OR stroke OR strokes)) OR (TITLE-ABS((brain OR cerebral OR intracranial OR intracerebral) W/2 (bleed* OR

embolism* OR hemiparesis OR hemorrhage* OR infarct* OR infract* OR injur* OR isch?emi* OR thrombo* OR "vascular accident*")))) AND (TITLE-ABS(arm OR arms OR axilla* OR elbow* OR finger* OR forearm* OR hand OR hands OR metacarpus OR shoulder* OR thumb* OR "upper extremit*" OR "upper limb*" OR wrist*)) AND (TITLE-ABS((touch* OR tactile OR cutaneous* OR skin OR haptic* OR sensation* OR sense OR senses OR sensory OR somatosensory OR perception*) W/6 (deficit* OR hemiplegia* OR impairment* OR monoplegia* OR dysfunction OR dysfunctions OR assessment* OR detect* OR evaluation* OR examination* OR index OR indexes OR instrument OR instruments OR measure* OR outcome* OR questionnaire* OR scale OR scales OR score OR scores OR survey OR surveys OR test OR tests)))) OR ((TITLE-ABS((touch* OR tactile OR cutaneous* OR skin OR haptic* OR sensation* OR sense OR senses OR sensory OR somatosensory OR perception*) W/2 (deficit* OR hemiplegia* OR impairment* OR monoplegia* OR dysfunction OR dysfunctions OR assessment* OR detect* OR evaluation* OR examination* OR index OR indexes OR instrument OR instruments OR measure* OR outcome* OR questionnaire* OR scale OR scales OR score OR scores OR survey OR surveys OR test OR tests))) AND (TITLE(apople* OR "cerebral accident*" OR "cerebrovascular accident*" OR poststroke OR stroke OR strokes))) AND (LIMIT-TO (LANGUAGE,"English"))

CINAHL Plus with Full Text

#	Query	Results
S24	S4 AND S7 AND S22 Limiters - English Language	291
S23	S4 AND S7 AND S22	294
S22	S18 OR S21	6,843
S21	S19 AND S20	718
S20	TI apople* OR "cerebral accident*" OR "cerebrovascular accident*" OR poststroke OR stroke OR strokes	65,505
S19	TI ((touch* OR tactile OR cutaneous* OR skin OR haptic* OR sensation* OR sense OR senses OR sensory OR somatosensory OR perception*) N6 (deficit* OR hemiplegia* OR impairment* OR monoplegia* OR dysfunction OR dysfunctions OR assessment* OR detect* OR evaluation* OR examination* OR index OR indexes OR instrument OR instruments OR measure* OR outcome* OR questionnaire* OR scale OR scales OR score OR scores OR survey OR surveys OR test OR tests)) OR AB ((touch* OR tactile OR cutaneous* OR skin OR haptic* OR sensation* OR sense OR senses OR sensory OR somatosensory OR perception*) N6 (deficit* OR hemiplegia* OR impairment* OR monoplegia* OR dysfunction OR dysfunctions OR	54,789

	assessment* OR detect* OR evaluation* OR examination* OR index OR indexes OR instrument OR instruments OR measure* OR outcome* OR questionnaire* OR scale OR scales OR score OR scores OR survey OR surveys OR test OR tests))	
S18	S11 AND S17	6,165
S17	S12 OR S13 OR S14 OR S15 OR S16	1,161,654
S16	(MM "Patient Assessment")	6,104
S15	(MH "Surveys") OR (MH "Data Collection Methods")	166,975
S14	(MH "Somatosensory Disorders")	618
S13	(MH "Hemiplegia")	6,754
S12	(MM "Diagnosis+") OR (MH "Clinical Assessment Tools+") OR (MH "Skin Tests+") OR (MH "Disability Evaluation+")	1,010,262
S11	S8 OR S9 OR S10	34,495
S10	(MM "Perception")	15,421
S9	(MH "Skin")	14,991
S8	(MH "Touch")	4,451
S7	S5 OR S6	218,493
S6	TI (arm OR arms OR axilla* OR elbow* OR finger* OR forearm* OR hand OR hands OR metacarpus OR shoulder* OR thumb* OR "upper extremit*" OR "upper limb*" OR wrist*) OR AB (arm OR arms OR axilla* OR elbow* OR finger* OR forearm* OR hand OR hands OR metacarpus OR shoulder* OR thumb* OR "upper extremit*" OR "upper limb*" OR wrist*)	208,276
S5	(MH "Upper Extremity+")	45,512
S4	S1 OR S2 OR S3	166,504
S3	TI ((brain OR cerebral OR intracranial OR intracerebral) N2 (bleed* OR embolism* OR hemiparesis OR hemorrhage* OR infarct* OR infract* OR injur* OR isch?emi* OR thrombo* OR "vascular accident*")) OR AB ((brain OR cerebral OR	48,834

	intracranial OR intracerebral) N2 (bleed* OR embolism* OR hemiparesis OR hemorrhage* OR infarct* OR infract* OR injur* OR isch?emi* OR thrombo* OR "vascular accident*"))	
S2	TI (apople* OR "cerebral accident*" OR "cerebrovascular accident*" OR poststroke OR stroke OR strokes) OR AB (apople* OR "cerebral accident*" OR "cerebrovascular accident*" OR poststroke OR stroke OR strokes)	111,826
S1	(MH "Stroke+")	78,211

PsycInfo

#	Query	Results
S16	S11 OR S14<br>Limiters - English	511
S15	S11 OR S14	516
S14	S12 AND S13	332
S13	TI apople* OR "cerebral accident*" OR "cerebrovascular accident*" OR poststroke OR stroke OR strokes	18,254
S12	TI ((touch* OR tactile OR cutaneous* OR skin OR haptic* OR sensation* OR sense OR senses OR sensory OR somatosensory OR perception*) N2 (deficit* OR hemiplegia* OR impairment* OR monoplegia* OR dysfunction OR dysfunctions OR assessment* OR detect* OR evaluation* OR examination* OR index OR indexes OR instrument OR instruments OR measure* OR outcome* OR questionnaire* OR scale OR scales OR score OR scores OR survey OR surveys OR test OR tests)) OR AB ((touch* OR tactile OR cutaneous* OR skin OR haptic* OR sensation* OR sense OR senses OR sensory OR somatosensory OR perception*) N2 (deficit* OR hemiplegia* OR impairment* OR monoplegia* OR dysfunction OR dysfunctions OR assessment* OR detect* OR evaluation* OR examination* OR index OR indexes OR instrument OR instruments OR measure* OR outcome* OR	38,367

	questionnaire* OR scale OR scales OR score OR scores OR survey OR surveys OR test OR tests))	
S11	S4 AND S5 AND S10	293
S10	S8 OR S9	74,744
S9	TI ((touch* OR tactile OR cutaneous* OR skin OR haptic* OR sensation* OR sense OR senses OR sensory OR somatosensory OR perception*) N6 (deficit* OR hemiplegia* OR impairment* OR monoplegia* OR dysfunction OR dysfunctions OR assessment* OR detect* OR evaluation* OR examination* OR index OR indexes OR instrument OR instruments OR measure* OR outcome* OR questionnaire* OR scale OR scales OR score OR scores OR survey OR surveys OR test OR tests)) OR AB ((touch* OR tactile OR cutaneous* OR skin OR haptic* OR sensation* OR sense OR senses OR sensory OR somatosensory OR perception*) N6 (deficit* OR hemiplegia* OR impairment* OR monoplegia* OR dysfunction OR dysfunctions OR assessment* OR detect* OR evaluation* OR examination* OR index OR indexes OR instrument OR instruments OR measure* OR outcome* OR questionnaire* OR scale OR scales OR score OR scores OR survey OR surveys OR test OR tests))	74,596
S8	S6 AND S7	286
S7	DE "Sensorimotor Measures" OR DE "Evaluation" OR DE "Disability Evaluation" OR DE "Evaluation Criteria" OR MM "Measurement"	70,264
S6	DE "Tactual Perception" OR MM "Skin (Anatomy)" OR MM "Perception"	25,009
S5	TI (arm OR arms OR axilla* OR elbow* OR finger* OR forearm* OR hand OR hands OR metacarpus OR shoulder* OR thumb* OR "upper extremit*" OR "upper limb*" OR wrist*) OR AB (arm OR arms OR axilla* OR elbow* OR finger* OR forearm* OR hand OR hands OR metacarpus OR shoulder* OR thumb* OR "upper extremit*" OR "upper limb*" OR wrist*)	145,826
S4	S1 OR S2 OR S3	72,100

S3	TI ((brain OR cerebral OR intracranial OR intracerebral) N2 (bleed* OR embolism* OR hemiparesis OR hemorrhage* OR infarct* OR infract* OR injur* OR isch?emi* OR thrombo* OR "vascular accident*")) OR AB ((brain OR cerebral OR intracranial OR intracerebral) N2 (bleed* OR embolism* OR hemiparesis OR hemorrhage* OR infarct* OR infract* OR injur* OR isch?emi* OR thrombo* OR "vascular accident*"))	37,755
S2	TI (apople* OR "cerebral accident*" OR "cerebrovascular accident*" OR poststroke OR stroke OR strokes) OR AB (apople* OR "cerebral accident*" OR "cerebrovascular accident*" OR poststroke OR stroke OR strokes)	38,405
S1	DE "Cerebrovascular Accidents"	23,342

ProQuest Dissertations & Theses Global

Set#: S1
Searched for: noft(apople* OR "cerebral accident*" OR "cerebrovascular accident*" OR poststroke OR stroke OR strokes) OR noft((brain OR cerebral OR intracranial OR intracerebral) NEAR/2 (bleed* OR embolism* OR hemiparesis OR hemorrhage* OR infarct* OR infract* OR injur* OR isch?emi* OR thrombo* OR "vascular accident*"))
Databases: ProQuest Dissertations & Theses Global
Results: 22214

Set#: S2
Searched for: noft(arm OR arms OR axilla* OR elbow* OR finger* OR forearm* OR hand OR hands OR metacarpus OR shoulder* OR thumb* OR "upper extremit*" OR "upper limb*" OR wrist*)
Databases: ProQuest Dissertations & Theses Global
Results: 181693

Set#: S3
Searched for: noft((touch* OR tactile OR cutaneous* OR skin OR haptic* OR sensation* OR sense OR senses OR sensory OR somatosensory OR perception*) NEAR/6 (deficit* OR hemiplegia* OR impairment* OR monoplegia* OR dysfunction OR dysfunctions OR assessment* OR detect* OR evaluation* OR examination* OR index OR indexes OR instrument OR instruments OR measure* OR outcome* OR questionnaire* OR scale OR scales OR score OR scores OR survey OR surveys OR test OR tests))
Databases: ProQuest Dissertations & Theses Global
Results: 50035

Set#: S4
Searched for: S1 AND S2 AND S3

Databases: ProQuest Dissertations & Theses Global
These databases are searched for part of your query.
Results: 94

Set#: S5
Searched for: noft((touch* OR tactile OR cutaneous* OR skin OR haptic* OR sensation* OR sense OR senses OR sensory OR somatosensory OR perception*) NEAR/2 (deficit* OR hemiplegia* OR impairment* OR monoplegia* OR dysfunction OR dysfunctions OR assessment* OR detect* OR evaluation* OR examination* OR index OR indexes OR instrument OR instruments OR measure* OR outcome* OR questionnaire* OR scale OR scales OR score OR scores OR survey OR surveys OR test OR tests))
Databases: ProQuest Dissertations & Theses Global
Results: 24223

Set#: S6
Searched for: ti(apople* OR "cerebral accident*" OR "cerebrovascular accident*" OR poststroke OR stroke OR strokes)
Databases: ProQuest Dissertations & Theses Global
Results: 4304

Set#: S7
Searched for: S5 AND S6
Databases: ProQuest Dissertations & Theses Global
These databases are searched for part of your query.
Results: 94

Set#: S8
Searched for: S4 OR S7
Databases: ProQuest Dissertations & Theses Global
These databases are searched for part of your query.
Results: 159

Set#: S9
Searched for: (S4 OR S7) AND la.exact("ENG")
Databases: ProQuest Dissertations & Theses Global
These databases are searched for part of your query.
Results: 151ProQuest Dissertations & Theses Global
Set#: S1
Searched for: noft((brain* OR cerebr* OR cerebell* OR intracran* OR intracerebral) NEAR/2 (injur* OR ischemi* OR ischaemic* OR infarct* OR thrombo* OR emboli* OR occlus* OR vasc*)) OR noft((disc OR discs OR disk OR disks) NEAR/2 (degeneration OR pain)) OR noft((spinal OR spine OR vertebra OR lumbar) NEAR/2 (pain OR injur*)) OR noft(physical* NEAR/2 (disabilit* OR disable* OR handicapped OR impair*)) OR noft(stroke* OR amputat* OR amputee* OR amyloidosis OR cancer patient* OR chronic back pain OR cerebrovasc* OR chronic inflammatory demyelinating polyneuropathy OR cryoglobulinemia OR cva* OR guillain barre OR limb loss OR lupus OR multifocal motor neuropathy OR multiple sclerosis OR "nervous system immune" OR neurologic autoimmune OR parkinson* OR peripheral neuropathy OR rheumatoid arthritis OR sciatica OR vasculitis)
Databases: ProQuest Dissertations & Theses Global

Results: 63498

Set#: S2
Searched for: noft(mentor* or mentee* or peer*)
Databases: ProQuest Dissertations & Theses Global
Results: 63704

Set#: S3
Searched for: noft("job accommodation*" or employment or "return to work" or "returning to work" or "job retention" or "work adjustment*" or vocational or "job reintegration" or work or employability)
Databases: ProQuest Dissertations & Theses Global
Results: 794915

Set#: S4
Searched for: S1 AND S2 AND S3
Databases: ProQuest Dissertations & Theses Global

Results: 144
